# Association of ADHD symptoms, depression and suicidal behaviors with anxiety in Chinese medical college students

**DOI:** 10.1186/s12888-020-02555-7

**Published:** 2020-04-22

**Authors:** Yanmei Shen, Yaru Zhang, Bella Siu Man Chan, Fanchao Meng, Tingyu Yang, Xuerong Luo, Chunxiang Huang

**Affiliations:** 1 Department of Psychiatry, The Second Xiangya Hospital, Central South University, Changsha, 410011 Hunan China; 2 Mental Health Institute of Central South University, China National Clinical Research Center on Mental Disorders (Xiangya), China National Technology Institute on Mental Disorders, Hunan Technology Institute of Psychiatry, Hunan Key Laboratory of Psychiatry and Mental Health, Changsha, 410011 Hunan China; 3 The Department of Educational and Counselling Psychology, and Special Education, The University of British Columbia, Vancouver, Canada

**Keywords:** Anxiety, ADHD, Suicidal behaviors, Risk factors, Medical college students

## Abstract

**Background:**

Anxiety is one of the most common psychiatric disorder and imposes a great burden on both the individual and the society. Previous studies indicate a high comorbidity of anxiety disorders and Attention Deficit Hyperactivity Disorder (ADHD). However, few studies have examined the comorbidity of anxiety and ADHD among medical college students in mainland China. This study aimed to examine the prevalence of anxiety and the associated risk factor of anxiety disorder as well as to explore the association between ADHD symptoms, depression, suicidal behaviors and anxiety.

**Methods:**

A cross-sectional design was employed among 4882 medical college students who were recruited and enrolled with convenience sampling. Self-reported demographic information and clinical characteristics were collected online on a computer or through a social media app named Wechat.

**Results:**

The prevalence of anxiety in this study was 19.9%. Students with anxiety were more likely to have a poor relationship with parents, be of Han nationality, have smoking or drinking habits, have an extensive physical disorder history and have engaged in suicidal behaviors. The independent risk factors for anxiety were: smoking, physical disorder history, suicidal ideations, suicide attempts, inattention and hyperactivity. Significant associations were observed between anxiety and depression, inattention, hyperactivity, suicide plans and suicide attempts.

**Conclusions:**

Nearly one in five medical students suffered from anxiety. The findings of this study indicate the importance of addressing both anxiety and ADHD symptoms in order to better promote mental health and the well-being of medical students as well as reduce suicidal behaviors.

## Background

Anxiety is one of the most common psychiatric disorder and inflicts a great burden on the individual and the society [1]. Anxiety disorder encompasses a class of maladies that include Generalized Anxiety Disorder, Panic Disorder and Social Phobia. These disorders are all characterized by excessive fear, anxiety and related behavioral disturbances [2]. The adverse effects of anxiety disorders could result in low life satisfaction for individuals [3] and a productivity loss for society [4].

At the beginning of the twenty-first century (in February, 2001 and April, 2003), the US National Comorbidity Survey Replication (NCS-R) reported that 28% of individuals with anxiety disorders would live with the disorder for their lifetimes [5]. Baumeister and Harter reported that the 12-month prevalence of anxiety varied from 5.6 to 18.1% [6]. Bayram & Bilgel found that higher rates of anxiety were observed among college students, particularly medical students, due to the tremendous pressures of their studies [7]. The prevalence rate reminded high for this specific group of population worldwide [8–10]. According to a study conducted by Zeng et al., the prevalence of anxiety among nursing students in Chinese vocational colleges was41.7% [11], and the prevalence of anxiety among Chinese medical students was 47.3% [12]. Alvi et al. suggested that high academic stress in medical schools as well as the extended contact with suffering patients may have an influence on the mental health of medical students [13]. Conversely, the anxiety symptoms of medical students may also generate negative influences on their professional performance with patients, for example, they may have less enthusiasm and compassion in caring for patients [14]. Therefore, studies examining anxiety disorders among medical students is of clinical significance for it may generate findings that could help students to improve their academic performance and achieve occupational success, as well as reduce the social burden of anxiety and its related costs.

Anxiety and depression are the most common comorbidity among mental health disorders [15, 16]. Approximately 85% of individuals with depression have a comorbid anxiety disorder. Similarly, up to 90% of individuals with anxiety also experience depression [17]. Anxiety and depression are also common psychological problems among medical students [18, 19]. A cross-sectional study from a medical school in Ethiopia showed that that 30.1 and 51.3% of medical students suffered from anxiety and depression, while 21.2% of them had a comorbidity of anxiety and depression [20]. Although the prevalence of depression and anxiety is remarkably high among Chinese medical students [21], few studies examined the co-morbidity of the two disorders in this population. Quite a few theories have suggested that depression has been found to increase the risk of suicide [22, 23]. However, there has been disagreement as to whether comorbid anxiety symptoms or anxiety disorders among depressed individuals puts those individuals at risk for suicide. A longitudinal study of data from the Netherlands Mental Health Survey shows that after controlling for a wide range of mental conditions, comorbid anxiety and depression increased the risk of suicide attempts [24]. Other studies pointed out that several but not all anxiety disorders (e.g. GAD) were risk factors for suicidal behaviors in the general population [25, 26].

Some studies [27, 28] indicate a high comorbidity of anxiety disorders and Attention Deficit Hyperactivity Disorder (ADHD). For instance, Pliszka et al. [29] reported that approximately among 25% of children with ADHD highly comorbid with anxiety disorders, the high comorbidity might result from the overlap of symptoms that characterize the two disorders. Further, some research suggested that the underlying neurobiological pathologies of ADHD and anxiety might contribute to their comorbidity. Specifically, a reduction of hippocampal and prefrontal cortex dopamine gating were observed be related to amygdala-based anxiety in children with ADHD [30]. However, that precise nature of the pertinent pathologies remains unclear.

Several studies have examined the prevalence of anxiety disorders and their associations with ADHD, depression and suicidal behaviors among Chinese children/adolescents [31–33]. However, as previously mentioned, to our knowledge, very few examined the comorbidity of anxiety and ADHD among adults in mainland China. Therefore, the aims of this study were: (1) to examine the prevalence and associated risk factors of anxiety disorders among medical college students in mainland China, and (2) to explore the association between ADHD symptoms, depression, suicidal behaviors and anxiety.

## Methods

### Participants

A total of 5004 medical college students were invited by a cross-sectional design between January and March of 2018. Convenience sampling selected four classes with 165 medical students at Changsha Health Vocational College; 50 classes with 4759 students at Yiyang Medical College, and; two classes with 80 students at Hunan University of Chinese Medicine. Changsha Health Vocational College and Yiyang Medical College provide three-year undergraduate programs. As a comprehensive medical university, the Hunan University of Chinese Medicine provides a four-year undergraduate medical program.

College instructors were trained how to direct students to fill the scales. All of the participants were informed that they could choose to fill the questionnaires out either online or through a widely used social media app (WeChat, Tencent Inc., China). All of the participants were informed of their rights to either participate or decline to participate or withdraw at any time. One hundred and ten students declined to participate, and 12 were excluded due to incomplete or missing data in their questionnaires. A total of 4882 participants were enrolled in the study. The study protocol was approved by the Ethics Committee of the Second Xiangya Hospital at Central South University in Changsha, China. All enrolled students signed an official written consent form that had been approved by the Ethics Committee.

### Clinical measures

The socio-demographic data included gender, community, single child or child with siblings, good relationship with mother and father, smoking, alcohol use, nationality, family income, parents’ education levels, mental disorder history, physical disorder history, family history of mental disorders, handedness, age and body mass index (BMI).

The 20-item Self-Rating Anxiety Scale (SAS) and the Self-Rating Depression Scale (SDS) [34, 35] were employed to measure anxiety and depression symptoms over the previous seven days. The SAS and SDS are widely used self-rating scales and are reported to have good psychometric properties in Chinese populations [36, 37]. Participants were regarded as having anxiety disorders and depression when their total scores exceeded 50 on the SAS and 53 on the SDS [38]. Participants with anxiety were assigned to the anxiety group and participants without anxiety were assigned to the control group.

Suicidal behaviors consisted of suicidal ideations, suicide plans and suicide attempts [39]. Suicidal ideations referred to passive thoughts of wishing for death. Suicide plans involved making plans or preparations for a suicide attempt. Suicide attempts were defined as potentially self-injuring actions that individuals engaged in with an intention to die [39, 40]. The questions asked about suicidal ideations, suicide plans and suicide attempts included: “Have you ever had any thoughts of committing suicide? Have you ever made a suicide plan? Have you ever tried committing suicide?” If an affirmative answer was given to the questions regarding suicide attempts, further questions were asked about the frequency and methods of suicide attempts.

The 18-item World Health Organization (WHO) Adult ADHD Self-report Scale v 1.1 Symptom Checklist (ASRS) was employed to assess current ADHD symptoms. The measure has been proven to have strong psychometric properties for discerning ADHD in a representative sample (Kessler et al., 2007). The ASRS scale consisted of two subscales: a 9-item inattention subscale (ASRSA) and a 9-item hyperactivity-impulsivity subscale (ASRSH). Each item was rated on a five-point Likert scale: 0 for never, 1 for rarely, 2 for sometimes, 3 for often, and 4 for very often. The total score for each subscale (ASRSA and ASRSH) ranged from 0 to 36. Individuals with scores of 17 or higher on either subscale were considered as positive for that subscale [41]. The Chinese version of the ASRS was proved to have a satisfactory psychometric property in [42].

### Data analysis

The Kolmogorov-Smirnov one-sample test was utilized to examine the normal distribution of continuous data. BMI, age, SDS, and SAS did not fit the normal distribution. Therefore, a non-parameter Mann-Whitney U test was utilized to compare the differences between the anxiety and control groups. A chi-squared test was employed to analyze the categorical variables. Multivariate logistic regression analyses were employed for variables showing a statistical trend (*P* < 0.1) to identify which of those variables were independently correlated with anxiety. Relationships between the variables were measured with Spearman product moment correlation coefficients. In addition, a stepwise multiple regression analysis was utilized to investigate the relationships between SAS and variables showing a statistical trend (*P* < 0.1). All statistical analyses were calculated with SPSS (Version 22.0; IBM, Inc., Chicago, Illinois), with the significance level set at 0.05.

## Results

The prevalence of anxiety among the participants in this study was 19.9% (972/4882). There were no significant differences between participants with and without anxiety for BMI, gender, single child, community, family income, right-handedness, parents’ education level, mental disorder history, and family history of mental disorder (*P* > 0.05, Table 1). Participants with anxiety were significantly younger (18.6 ± 1.1) than participants without anxiety (18.8 ± 1.1) (*P* < 0.001, Table 1). In addition, as demonstrated by Table 1, participants with anxiety were more likely to have a poor relationship with mother or father; be of Han nationality; smoke or drink alcohol; have a physical disorder history, and have had suicidal ideations, planned suicides, or made suicide attempts (*P* < 0.05).

**Table 1:** Demographics and clinical characteristics between Anxiety and control groups

Variable	control *n* = 3910		Anxiety *n* = 972		χ^2^/Z	p	OR(95% CI)
Age (years), mean (SD)	18.8 (1.1)		18.6 (1.1)		−4.739	0.000***	
BMI (kg m^−2^), mean (SD)	20.1 (2.4)		20.1 (2.6)		−0.586	0.558	
Gender							
Males, n (%)	431	11.00%	106	10.90%	0.011	0.916	1.012 (0.808–1.268)
Females, n (%)	3479	89.00%	866	89.10%			
Single child, n (%)	991	25.30%	258	26.50%	0.587	0.444	1.064 (0.907–1.248)
Community							
Rural	2820	72.10%	700	72.00%	0.004	0.947	1.005 (0.860–1.176)
Urban	1090	27.90%	272	28.00%			
Good relationship with mother, n (%)	3811	97.50%	912	93.80%	32.752	0.000***	0.395 (0.284–0.549)
Good relationship with father, n (%)	3747	95.80%	887	91.30%	33.808	0.000***	0.454 (0.346–0.596)
Nationality							
Others	517	13.20%	105	10.80%	4.101	0.043*	1.258 (1.007–1.572)
Han	3393	86.80%	867	89.20%			
Smoking, n (%)	134	3.40%	78	8.00%	39.614	0.000***	2.459 (1.842–3.281)
Alcohol, n (%)	580	14.80%	217	22.30%	31.982	0.000***	1.650 (1.385–1.965)
Family income /year (yuan)							
Less than 30,000, n (%)	1624	41.50%	431	44.30%	3.601	0.308	
30,000~50,000, n (%)	1336	34.20%	328	33.70%			
50,000~70,000, n (%)	549	14.00%	119	12.20%			
More than 70,000, n (%)	401	10.30%	94	9.70%			
Father’s education level							
Junior middle school and below, n (%)	2539	64.90%	619	63.70%	0.701	0.704	
High School or Technical School, n (%)	1005	25.70%	255	26.20%			
College or University and above, n (%)	366	9.40%	98	10.10%			
Mother’s education level							
Junior middle school and below, n (%)	2865	73.30%	691	71.10%	1.89	0.389	
High School or Technical School, n (%)	811	20.70%	219	22.50%			
College or University and above, n (%)	234	6.00%	62	6.40%			
Right-handedness, n (%)	3370	86.20%	855	88.00%	2.103	0.147	1.171 (0.946–1.450)
Physical disorder history, n (%)	224	5.70%	102	10.50%	28.363	0.000***	1.929 (1.509–2.466)
Mental disorder history, n (%)	27	0.70%	11	1.10%	1.962	0.161	1.646 (0.814–3.330)
Family history of mental disorder, n (%)	77	2.00%	17	1.70%	0.2	0.655	0.886 (0.522–1.505)
Suicidal ideation, n (%)	829	21.20%	460	47.30%	273.388	0.000***	3.339 (2.881–3.869)
Suicide plans, n (%)	193	4.90%	178	18.30%	198.377	0.000***	4.318 (3.473–5.367)
Suicide attempts, n (%)	380	9.70%	302	31.10%	295.294	0.000***	4.187 (3.525–4.974)

Note: *p < 0.05; ***p < 0.001

Table 2 shows that participants with anxiety scored significantly higher on the ASRS attention subscale, ASRS hyperactivity subscale, ASRS total scale and SDS (*p* < 0.001), with an effect size ranging from − 0.35 to − 0.64.

**Table 2:** ADHD and SDS scores in Anxiety and control groups

Variable	control n = 3910		Anxiety n = 972		χ^2^/Z	p	OR(95% CI)	Effect size
ASRSA, mean (SD)	12.2 (4.9)		15.8 (4.8)		−19.235	0.000***		−0.35
ASRSA≥17	727	18.60%	451	46.40%	328.773	0.000***	3.790 (3.263–4.402)	
ASRSH, mean (SD)	9.0 (4.3)		13.2 (4.6)		−23.668	0.000***		−0.43
ASRSH≥17	142	3.60%	228	23.50%	436.817	0.000***	8.132 (6.501–10.171)	
ASRST, mean (SD)	21.3 (8.3)		29.0 (8.4)		−23.444	0.000***		−0.42
ASRST≥34	767	19.60%	494	50.80%	395.724	0.000***	4.235 (3.651–4.913)	
SDS, mean (SD)	44.6 (8.7)		57.9 (7.3)		−36.322	0.000***		−0.64
SDS>53	794	20.30%	768	79.00%	1233.043	0.000***	14.774 (12.428–17.563)	

Note: *ASRS* Adult ADHD Self-Report Scale, *ASRSA* Adult ADHD Self-Report Scale-Inattention subscale, *ASRSH* Adult ADHD Self-Report Scale-Hyperactivity subscale, *ASRST* Adult ADHD Self-Report Scale-Hyperactivity total score, *SDS* Self—Rating Depression Scale, ***p < 0.001

The variables of physical disorder history (OR: 1.429; 95% CI: 1.056–1.934; *P* = 0.021), suicide plans (OR: 1.481; 95% CI,:1.081–2.028; *P* = 0.014), suicide attempts (OR:1.748; 95% CI: 1.327–2.304; *P* < 0.001), ASRSA (OR:1.558; 95% CI: 1.286–1.887; *P* < 0.001), ASRSH (OR:2.871; 95% CI: 2.178–3.784; *P* < 0.001), and depression (OR:10.552; 95% CI: 8.804–12.646; *P* < 0.001) remained significant after adjusting for confounders in the multivariate logistic regression analysis (Table 3).

**Table 3:** Multivariate analysis for variables associated with anxiety among medical students

	B	S.E.	Wald	df	Sig.	Exp(B)	95% C.I.for EXP(B)	
Variable							Lower	Upper
Good relationship with mother (yes)	−.299	.237	1.592	1	.207	.742	.466	1.180
Good relationship with father (yes)	−.116	.197	.345	1	.557	.891	.605	1.311
Han	.247	.136	3.302	1	.069	1.280	.981	1.670
Smoking (yes)	.404	.207	3.795	1	.051	1.497	.998	2.247
Drinking (yes)	.084	.119	.500	1	.480	1.088	.861	1.375
Physical disorder history (yes)	.357	.154	5.364	1	021*	1.429	1.056	1.934
Suicidal ideation (yes)	.193	.117	2.721	1	.099	1.213	.964	1.526
Suicide plans (yes)	.393	.160	5.991	1	.014*	1.481	1.081	2.028
Suicide attempts (yes)	.559	.141	15.738	1	.000***	1.748	1.327	2.304
ASRSA (yes)	.443	.098	20.605	1	.000***	1.558	1.286	1.887
ASRSH (yes)	1.055	.141	56.032	1	.000***	2.871	2.178	3.784
Depression (yes)	2.356	.092	650.467	1	.000***	10.552	8.804	12.646

Note: *ASRSA* Adult ADHD Self-Report Scale-Inattention subscale, *ASRSH* Adult ADHD Self-Report Scale-Hyperactivity subscale, *SDS* Self—Rating Depression Scale; *p < 0.05; ***p < 0.001

In addition, a Pearson correlation analysis among participants with anxiety (SAS > 50) showed significant correlations between the SAS total score and the following parameters: good relationship with father (*r* = − 0.109, df = 972, *p* = 0.001); depression (*r* = − 0.427, df = 972, *p* < 0.001);suicidal ideations(*r* = 0.144, df = 972, *p* < 0.001); suicide plans(*r* = 0.183, df = 972, *p* < 0.001), and; suicide attempts(*r* = 0.170, df = 972, *p* < 0.001), ASRSA(*r* = 0.183, df = 972, *p* < 0.001), and ASRSH(*r* = 0.337, df = 972, *p* < 0.001). All significant differences passed the Bonferroni **corrections** (Bonferroni corrected *p* < 0.05/12 = 0.0042; all *p* ≤ 0.001). Finally, the stepwise regression showed significant associations between the SAS score and SDS (beta = 0.365, t = 12.582, *p* < 0.001); ASRSH (beta = 0.295, t = 8.434, *p* < 0.001), ASRSA (beta = − 0.097, t = − 2.767, *p* = 0.006); suicide plans (beta = 0.078, t = 2.713, *p* = 0.007) and good relationship with father (beta = − 0.057, t = − 2.011, *p* = 0.045).

## Discussion

Our study showed that the prevalence of anxiety disorders was 19.9% among medical college students in Hunan province, China, which is similar to the prevalence of anxiety disorders for medical students in the United States (20.3%) [43], but lower than that of medical students in western countries (27.5%) [10] and Australia (45.1%) [9], higher than the prevalence of medical students reported in another report in China (14.1%) [8]. In addition, results of this study is significantly higher than the global prevalence for anxiety disorders among general population [44]. There could be several reasons for this higher prevalence. Firstly, mental health problems are reported to be common among college students due to the stress and pressure of striving to succeed academically and having to make post-graduation plans [45]. Moreover, data from different countries showed a widespread prevalence of anxiety among undergraduate medical students [12, 19, 46–48]. Some studies suggested that this may in part stem from a lack of leisure time [48] during their medical education. In addition, the intense doctor-patient relationship might have adversely influenced students’ attitudes and led them to feel pessimistic about their future careers [49], especially given that so many conflicts between doctors and patients have been reported in China in recent years [50–53]. Another explanation for the prevalence of anxiety among medical students in this study could be the high proportion of female participants (89.0%) – previous studies have shown that females were more prone to suffer from anxiety disorders than males [54, 55]. In addition, our study found that students with Han nationality have higher levels of anxiety compared to students with other nationalities. Several reasons might contribute to this phenomenon. On the one hand, China has promulgated preferential policies for minorities, which was developed to support the development of minorities in economy, medical treatment, education, as well as other aspects [56]. For instance,, minorities have the advantage to obtain additional scores on the college entrance examination [57]. On the other hand, a large proportion of minorities in China engages in religious beliefs, in which they are reported to have a positive influence in increasing personal happiness and satisfaction [58]. In addition, Gonçalves et al. suggested that medical students with religious beliefs may relieve their anxiety symptoms in college life through religious support [59].

Anxiety disorders were found to be highly comorbid with ADHD in this study (50.8%), which is consistent with previous evidence [60, 61]. Schatz and Rostain [62] suggested that the deficits of early cognitive regulatory among individuals with ADHD may result in a failure to follow social norms and problems with peer relationships. This can in return result in emotional dysregulation, which characterizes anxiety disorders. One study suggested that the strong association between ADHD symptoms and anxiety symptoms might be explained by the similar brain structure pathologies that characterize the two disorders. For example, Levy [30] suggested that the nucleus accumbens is one of the crucial dopamine systems, so impairment to it would cause dysfunction in the three main dopaminergic circuits: the mesolimbic, mesocortical, and nigrostriatal circuits. In addition, they reported that dysfunction in the mesolimbic circuit might be related to the abnormal regulation of anxiety, while dysfunction in the mesocortical circuit might be related to attention deficits, and a poor nigrostriatal circuit might contribute to hyperactivity and emotional disturbances [30]. Nigg and Casey [63] assumed that ADHD symptoms may be involved in the cognitive control and affective responses**.** These authors suggested that deficits in various brain regions (e.g., the amygdala, the prefrontal cortex, the cerebellum) were associated with impulsive behavior or dysregulatory psychopathology, especially among individuals with ADHD. More studies are needed to explore the underlying pathomechanism that characterizes the association between anxiety and ADHD symptoms.

This study found that both inattention and hyperactivity symptoms were independently associated with anxiety symptoms and that hyperactivity symptoms had a higher OR than inattention symptoms. It is pertinent to note that previous research has assumed there are two possible distinct subtypes of ADHD −Attention Deficit Disorder without hyperactivity (ADD/WO) and Attention Deficit Disorder with hyperactivity (ADD/H). Children with ADD/WO were more likely to exhibit emotional disorders such as depression and anxiety, but demonstrated a lesser likelihood of serious behavioral problems than children with ADD/H [64]. However, some other studies reported inconsistent results with regard to this finding. For example, several studies found no significant difference in the anxiety and depression levels of individuals with the two subtypes of ADHD [65, 66]. The studies further found that individuals with the combined type of ADHA (ADHD/COM) had more severe symptoms of internalizing than the other sub-types [67].

Our study demonstrated a remarkably high correlation between anxiety and depression, which is consistent with previous evidence [68, 69]. There are several generally accepted explanations for the co-occurrence of anxiety and depression. The similarity between some symptoms of anxiety and depression offers one explanation. For example, both conditions are characterized by sleeping problems, restlessness and fatigue. Second, anxiety and depression might share common etiological factors. A high negative affectivity or emotionality is one latent risk factor for both conditions that is highly likely to contribute to their comorbidity [70, 71]. Third, twins’ studies reveal that genes likely account for the comorbidity [72]. Fourth, maladaptive cognition and information processing errors related to negative experiences of social events are associated with both anxiety and depression [73]. Finally, it has been suggested that the neural circuitry dysfunction involved in the emotional regulation of perceptions and behaviors exists in individuals with both anxiety and depression [74, 75]. For example, abnormalities in the responses of the amygdala have been observed in individuals with anxiety or mood disorders [75–77].

The findings of this study suggested that individuals with anxiety disorders were more likely to have suicidal behaviors than individuals without anxiety. In fact, some national organizations have listed anxiety disorders as a significant risk factor for suicidal behaviors [78]. One study suggested that it is the negative emotions related to anxiety that lead to suicidal behaviors [79], given that individuals usually attempt to end their lives in order to escape or gain relief from painful emotions [80, 81]. Furthermore, social avoidance, a characteristic feature of anxiety, is reported to lead to social isolation, a low quality of life, and disabilities, all of which might increase the risk of suicide [82–84]. Other studies suggested that an underlying biological pathology might be associated with acute suicidal acts. For example, one study found high corticotrophin releasing hormone levels in anxiety disorders, which might be associated with acute suicidal acts [85]. Finally, very few of the participants with anxiety reported a mental disorder history. This might indicate that they had a stigma towards mental disorders. Previous studies have suggested that medical students with mental health problems avoid seeking professional help because such problems are stigmatized and the students are embarrassed to have them [86, 87]. This might in lead to further deterioration and increase the risk of engaging in suicidal behaviors.

However, there were also some inconsistent findings. For example, a survey [88] revealed that individuals who suffered from panic disorder alone were not at high risk for suicide attempts, and a study done on a sample of outpatients with bipolar disorders showed no apparent association between suicide attempts and anxiety after controlling the mood disorder [89]. Another study inferred that higher levels of anxiety, hypochondriasis, agitation or fear of death might underlying protective factors against suicidality for inpatient groups with major depression disorders [90]. These discrepant findings might be explained by differences in study sample populations or sizes, assessment methods, methodologies, or data analyses processes [91]. On the other hand, as anxiety disorders consist of a spectrum of disorders, the types and severity levels of anxiety disorders should also be taken into consideration. For example, a meta-analysis of anxiety disorders showed that every type of anxiety disorder, excluding Obsessive-Compulsive Disorder, increases the risk of suicidal behaviors [84]. Further, patients with moderate or more severe anxiety symptoms were over three times the risk of having suicidality [92]. Further research is needed to investigate the underlying pathology between anxiety and suicide.

Several limitations to this study must be acknowledged. Firstly, as a large proportion of the participants are females, it might not representall medical students in China. Results obtained in the study are more relevant to female. The reason for the high proportion of females might be related to the following reasons. On one hand, the proportion of females in medical school in China is much higher than males (female: male = 7:3) [93]. On the other hand, this study utilized convenience sampling, therefore, classes including higher percentage of male students might not be included in the invited population. Future studies should have a balanced sample of males and females to increase the studies’ representativeness. Secondly, as the measurement tool used in this study was self-reported assessment scales, a bias may have resulted from participants’ stigma towards mental health conditions [94]. Thirdly, an absence of priori analysis is a limitation, so in future, researchers should conduct a priori analysis before conducting studies similar to the current study. Finally, as a cross-sectional study was employed for this study, its findings were insufficient to draw any conclusions about causal relationships between anxiety and other variables. Longitudinal designs are needed to explore those causal relationships.

## Conclusion

In summary, we found that nearly 1 in 5 medical students suffered from anxiety. Poor relationships with parents, being of Han nationality, smoking or alcohol habits, physical disorder history, depression and suicidal behaviors were associated with anxiety symptoms. Inattention and hyperactivity symptoms were independent risk factors for anxiety. The study revealed strong associations between anxiety symptoms and ADHD symptoms, depression and suicidal behaviors. This points to the importance of addressing ADHD symptoms among students with anxiety. It also highlights the importance of screening medical students for anxiety disorders in order to better promote the mental health and well-being of this population and better prevent suicidal behaviors.

## Data Availability

The datasets that were generated analyzed for the current study are not publicly available as the author does not have permission to share the data.

## Abbreviations

DSM-IV: Diagnostic and statistical manual of mental disorders, 4th edition
SDS: Zung’s self-rating depression scale
SAS: Self-rating anxiety scale
OR: Odds ratio
BMI: Body mass index
ASRS: Adult ADHD self-report scale
ASRSA: Adult ADHD self-report scale-inattention subscale
ASRSH: Adult ADHD self-report scale-hyperactivity subscale
ASRST: Adult ADHD self-report scale-hyperactivity total score
